# Bridging the educational gap: a pilot study of integrating patient-reported outcome into orthopedic residency training

**DOI:** 10.3389/fmed.2025.1635610

**Published:** 2026-01-06

**Authors:** Leyi Huang, Yao Chen, Zhuolin Zhong, Huangrong Zhu, Honghua Hu, Xiaoming Xu

**Affiliations:** 1 Department of Orthopedics, The Fourth Affiliated Hospital of School of Medicine, and International School of Medicine, International Institutes of Medicine, Zhejiang University, Yiwu, China; 2 Department of Dermatology, The Fourth Affiliated Hospital of School of Medicine, and International School of Medicine, International Institutes of Medicine, Zhejiang University, Yiwu, China; 3 Center for International Medical Education, The Fourth Affiliated Hospital of School of Medicine, International School of Medicine, and International Institutes of Medicine, Zhejiang University, Yiwu, China

**Keywords:** bone and joint surgery, orthopedic residency programs, patient-reported outcome (PRO), resident trainees, standardized training

## Abstract

**Background:**

Patient-reported outcome (PRO) is vital for assessing treatment efficacy, yet they have not been systematically incorporated into most medical curricula. This pilot study evaluated the feasibility and impact of a PRO curriculum on orthopedic and joint surgery residents’ knowledge and attitudes.

**Methods:**

Fifty orthopedic and joint surgery resident trainees undergoing standardized training at a teaching hospital from January 2025 to May 2025 were divided into two groups: the PRO teaching reform experimental group (Experimental group) and the traditional model teaching group (Control group). The PRO teaching reform experimental group (*n* = 25) received a structured PRO curriculum alongside standard training, while the traditional model teaching group (*n* = 25) received traditional standard training alongside remedial training. Both groups completed pre- and post-training PRO knowledge tests (0–100 scale) and Likert-scale feedback surveys. Despite their established clinical and research utility, a significant educational gap persists.

**Results:**

Despite similar baseline PRO knowledge (*p* = 0.45), the experimental group scored significantly higher on the post-training test than the control group (70.56 ± 5.89 vs. 58.68 ± 4.72, *p* < 0.001). Additionally, the experimental group expressed greater interest in the reformed curriculum (3.36 ± 0.86 vs. 2.88 ± 0.78, *p* = 0.04), higher satisfaction with the PRO content (3.72 ± 0.54 vs. 3.32 ± 0.63, *p* = 0.02), stronger agreement with the inclusion of PRO in medical intervention evaluations (3.76 ± 0.44 vs. 3.44 ± 0.59, *p* = 0.03), and a deeper impact on their medical cognition (3.80 ± 0.41 vs. 3.48 ± 0.59, *p* = 0.03), all with statistically significant differences.

**Conclusion:**

This pilot study demonstrated that the structured PRO curriculum was feasible to implement and significantly improved knowledge and attitudes among orthopedic residents. These findings support integrating PRO into surgical training and warrant larger trials to assess impact on clinical practice.

## 1 Introduction

Orthopedic surgery, particularly in the realm of elective procedures such as total joint arthroplasty (TJA), has increasingly shifted its focus from purely technical and radiological success to the enhancement of patient-centric outcomes. Quality of life, functional capacity, and overall well-being are now recognized as the definitive markers of therapeutic efficacy (1, 2). This paradigm shift necessitates robust methods to capture the patient’s subjective experience, a domain where clinician-reported metrics alone are insufficient. Patient-reported outcome (PRO)—rigorously developed and validated instruments completed directly by patients—have thus emerged as a methodological cornerstone in outcomes research and value-based healthcare (3, 4). Furthermore, PRO play a critical role in clinical practice, as evidenced by their formal incorporation into clinical guidelines and national programs, where they serve as pivotal evidence for evaluating surgical success and benchmarking healthcare performance (5, 6). Despite their established utility in clinical and research settings, a significant educational gap persists. Formal instruction on PRO—covering its conceptual foundations, psychometric properties, and clinical application—remains markedly absent from the core curricula of most medical schools and residency programs (7). This discrepancy creates a critical chasm: physicians are increasingly expected to utilize and interpret PRO data in practice, yet they receive little to no structured training. Evidence from Florentino et al. (7) substantiates this gap, demonstrating limited awareness and knowledge of PRO among medical students. Compounding this issue, the effective use of PRO requires understanding the broader context of patient care. For instance, social determinants of health significantly influence both the utilization of orthopedic procedures and subsequent PRO, as socioeconomic deprivation is associated with lower TJA rates and often diminished postoperative outcome improvements (8, 9). Therefore, a foundational understanding of PRO is essential not only for delivering patient-centered care but also for recognizing and addressing pervasive health disparities (8). The integration of PRO education is supported by studies showing its positive role in enhancing clinical reasoning and patient-centered care across diverse settings (10, 11); however, such integration in surgical training, particularly in orthopedics, remains notably lacking. To address this unmet educational need, we propose that integrating structured PRO instruction into standardized residency training represents a necessary step in modernizing surgical education. This pilot study was consequently designed to evaluate the feasibility and preliminary impact of a novel, structured PRO curriculum within an orthopedic surgery residency program. We hypothesized that this integrated educational intervention, compared to traditional training alone, would significantly improve residents’ PRO-related knowledge and foster more favorable attitudes toward their clinical implementation.

## 2 Methods

### 2.1 Study design and setting

**Figure 1:**
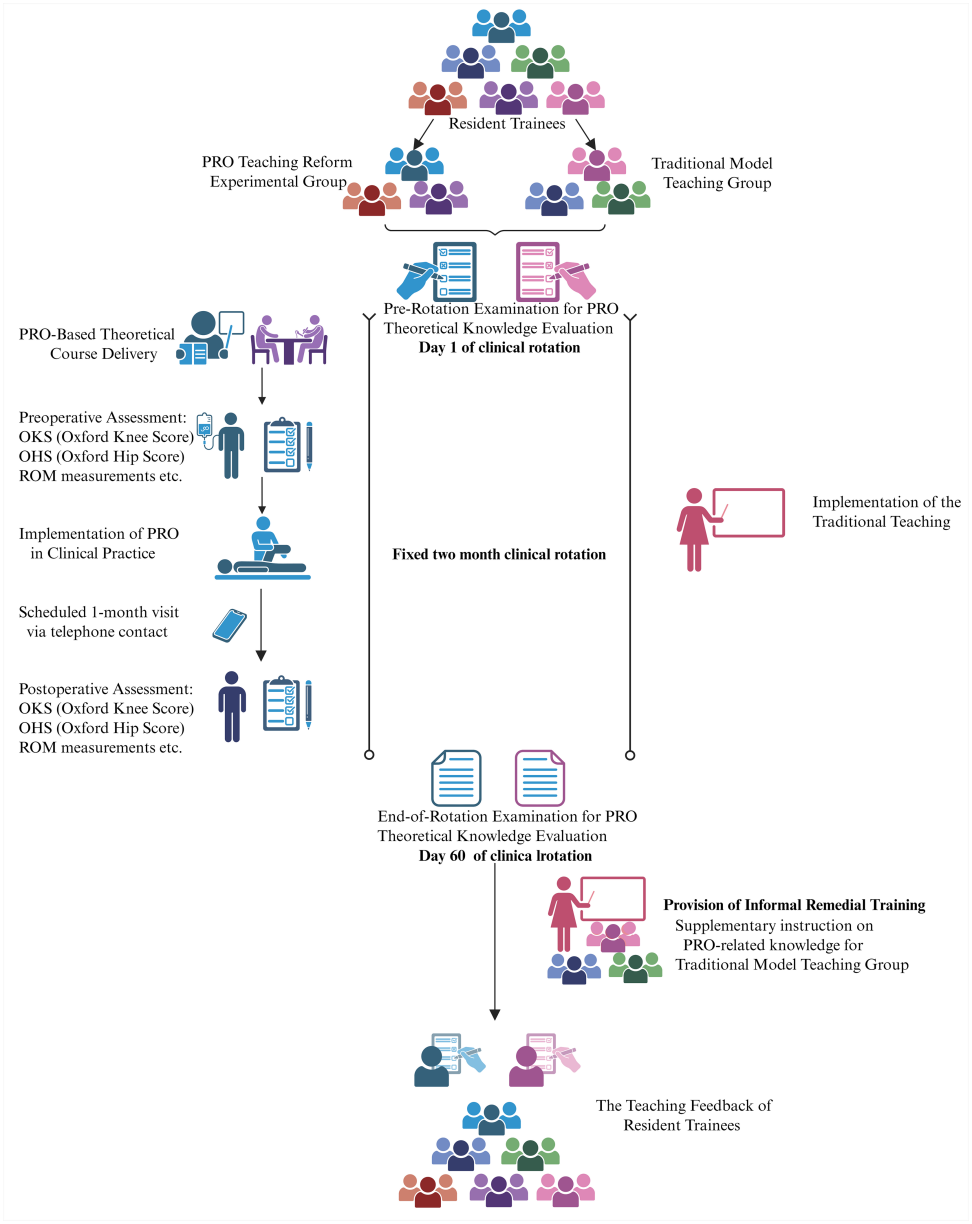
Flowchart of the study. Schematic of the 5-month pilot trial showing group allocation, the 2-month rotation-based intervention (PRO teaching reform experimental group vs. traditional model teaching group), and assessment timeline (pre/post-test and feedback).

This study was a pilot, quasi-experimental controlled trial conducted at a high-volume, 1,229-bed national teaching hospital, which is an accredited center for standardized residency training in China. The study was implemented over a five-month period, from January 2025 to May 2025. This five-month duration represented the total enrollment and data collection window for the study, accommodating the sequential entry of different resident cohorts based on their scheduled rotation blocks. A quasi-experimental design was employed whereby participants were assigned to either an experimental or control group based on their pre-determined, non-random clinical rotation schedules. The overall study design and participant flow are summarized in Figure 1. This design was chosen to evaluate the feasibility and preliminary impact of a structured PRO-integrated curriculum within the pragmatic constraints of the existing residency training framework. For each individual participant, the entire study participation—including the intervention (or control condition), pre-test, and post-test was completed within their single, fixed 2-month clinical rotation period.

### 2.2 Participants and recruitment

The participant pool consisted of orthopedic and joint surgery resident trainees from the 2023–2024 training cohort. A convenience sampling method was utilized to recruit participants. The inclusion criteria were: (1) being an enrolled resident trainee in orthopedic surgery, and (2) scheduled for a clinical rotation at the study site during the intervention period. There were no exclusion criteria. The final sample comprised 50 resident trainees.

The sample size of 50 (25 per group) was determined based on the goals of a pilot study, which prioritizes feasibility assessment and generation of preliminary effect size estimates over definitive hypothesis testing. This number was considered feasible given the size of the annual trainee cohort and available resources, including funding and instructional capacity. Although formal power calculation was not performed due to the absence of prior comparable data, this sample size is consistent with other pilot educational interventions reported in the literature (7).

### 2.3 Group allocation and baseline comparability

Participants were allocated to one of two groups based on their rotation schedules:

Experimental group (*n* = 25): This group received the novel PRO-integrated curriculum alongside the standard orthopedic training.
Control group (*n* = 25): This group continued with the traditional teaching model, which contained no PRO content.

To ensure group comparability at baseline, demographic and key professional characteristics were collected and compared. As detailed in Table 1, the two groups were well-matched in terms of age, sex, distribution of post-graduate years (PGY), number of prior orthopedic rotations, baseline PRO knowledge scores, and self-reported familiarity with PRO content. The absence of significant differences in these variables confirms the equivalence of the two groups at the outset of the study.

**Table 1:** Distribution of training years among resident trainees in the PRO teaching reform experimental group versus the traditional model teaching group.

Characteristics	PRO teaching reform experimental group	Traditional model teaching group
Age years	26.8 ± 1.5	27.1 ± 1.1
Sex male/female	12/13	13/12
PGY 1	6	6
PGY 2	16	16
PGY 3	3	3
Prior orthopedic rotations, *n*	2.4 ± 0.7	2.6 ± 0.9
Baseline PRO knowledge score	40.96 ± 4.55	42.04 ± 5.36
Familiarity with PRO content before taking this curriculum	0.76 ± 0.83	1.00 ± 0.86

### 2.4 Intervention: PRO-integrated curriculum

#### 2.4.1 Instructor qualification and training

The PRO curriculum for the experimental group was delivered by a multidisciplinary team of three instructors: two associate chief physicians from the Department of Orthopedics and one senior orthopedic nurse with over 10 years of clinical experience. Their qualifications were based on three pillars: (1) extensive clinical expertise in implementing outcome measures in daily practice; (2) active involvement as developers of the PRO curriculum on the Orthopedic Curriculum Committee, ensuring deep content knowledge; and (3) substantial prior experience in medical education. To standardize the delivery of the intervention, all instructors participated in a dedicated “train-the-trainer” session prior to the study commencement, focusing on the core content, teaching methods, and case discussions to be used.

#### 2.4.2 Curriculum development and theoretical delivery

The PRO-integrated curriculum was developed independently and from the ground up by the Orthopedic Curriculum Committee. The curriculum was developed through an expert consensus process guided by a committee of two medical education assessment specialists and two senior orthopedic surgeons. The curriculum was designed to augment the traditional standardized syllabus for orthopedic residency in China, which lacks any formal PRO instruction.

The theoretical component for the experimental group consisted of eight structured, 15-min modules delivered via multimedia presentations following standard didactic sessions. The modules sequentially covered: (1) Fundamentals of PRO: Conceptual background and global development. (2) Clinical value and significance in orthopedic practice. (3) Key considerations for clinical application. (4) Introduction to the Patient-Reported Outcome Measurement Information System (PROMIS). (5) PROMIS implementation methodologies with clinical case examples. (6) Clinical application of the Oxford Knee Score (OKS) for total knee arthroplasty (TKA) patients. (7) Clinical application of the Oxford Hip Score (OHS) for total hip arthroplasty (THA) patients. (8) Course summary and interactive question-and-answer session. OKS and OHS are disease-specific PRO instruments validated for patients undergoing total knee and total hip arthroplasty, respectively. They assess pain and function through 12-item questionnaires, with higher scores indicating better outcomes. The total additional teaching time for the PRO curriculum was 2 h, distributed over 2 months.

#### 2.4.3 Clinical practice integration

Beyond theoretical knowledge, the experimental group implemented standardized PRO protocols in clinical practice across the patient care continuum, the experimental group implemented PRO collection as a standardized component of routine clinical workflow for eligible patients under their care. The scope included all elective total knee arthroplasty (TKA) and total hip arthroplasty (THA) patients assigned to the trainees’ supervising attending physicians during their rotation.

##### 2.4.3.1 Preoperative assessment

Trainees collected OKS for TKA patients and OHS for THA candidates, in addition to routinely documenting pain scores, swelling severity, and range of motion (ROM). Standardized data collection forms were used, and trainees received direct supervision from the study instructors during initial assessments to ensure correct administration.

##### 2.4.3.2 Postoperative follow-up

At one-month post-surgery, trainees conducted follow-up visits via telephone or supervised outpatient evaluations, during which they re-administered the PRO measures (OKS/OHS) and performed standardized clinical assessments. All follow-up assessments followed a predefined protocol, and data were recorded in a dedicated study log under instructor supervision to maintain consistency.

This hands-on integration was designed to bridge theory and practice, allowing trainees to appreciate the utility of PRO in real-world clinical decision-making.

##### 2.4.3.3 Clinical data as educational tools

In this educational intervention, trainees administered and recorded OKS and OHS for patients under supervision. The collected real-world patient PRO data served primarily for clinical teaching discussions, such as during ward rounds or dedicated seminars, where trainees analyzed the relationship between PRO scores, clinical symptoms, and imaging findings in specific cases to deepen their understanding of PRO’s clinical significance. It is important to clarify that the primary endpoints of this study were the educational outcomes for the residents (knowledge acquisition and attitude change), not the clinical outcomes or PRO scores of the patient population themselves. Therefore, individual patient PRO data were not aggregated and reported as study outcome measures.

### 2.5 Control condition: traditional teaching model

The control group received the standard, traditional training as mandated by the national standardized curriculum for orthopedic residency. This curriculum focused on core surgical knowledge, clinical skills, and the assessment of objective clinical parameters. Specific content delivered during the study period included: principles of perioperative management in arthroplasty, surgical techniques, complication recognition, and the interpretation of imaging and laboratory findings. Clinical assessment emphasized objective measures such as wound inspection, neurologic examination, radiographic alignment, and measured range of motion. The instructional mode (multimedia presentations including PowerPoint and videos) was kept consistent with the experimental group to control for delivery variability. Crucially, the content for the control group was strictly limited to the traditional curriculum and contained absolutely no PRO-related concepts, instruments (e.g., PROMIS, OKS, OHS), or application examples. During ward rounds, control group trainees also assessed and documented objective measures like pain scores, swelling, and ROM, but they did so without any structured PRO framework or use of validated PRO instruments.

### 2.6 Outcome measures and assessment instruments

Two primary outcomes were assessed: (1) PRO-related knowledge, and (2) Trainee perceptions and feedback. Both groups underwent identical assessments at the beginning (pre-test) and end (post-test) of their rotation.

#### 2.6.1 Development of assessment questionnaires

Both assessment questionnaires were developed specifically for this study by the Orthopedic Curriculum Committee using an expert consensus process to ensure content validity. The item generation was informed by a comprehensive review of literature on PRO implementation and medical education (7, 12–14).

##### 2.6.1.1 PRO theoretical knowledge evaluation questionnaire

*Objective*: To quantitatively assess the breadth and depth of trainees’ understanding of core PRO concepts.

*Content and format*: The test consisted of 30 items: 10 multiple-choice questions (MCQs), 10 multiple-answer questions (MAQs), and 10 true/false questions. The questions comprehensively covered the key points of the curriculum, including PRO definition, clinical value, application precautions, PROMIS, OKS, and OHS.

*Scoring system and basis*: A standardized scoring key was established *a priori*. The total possible score was 100 points. The points were allocated as follows: each of the 10 multiple choice questions (MCQs) (single best answer) and 10 true/false questions was worth 3 points for a correct answer (totaling 60 points). For each of the 10 multiple-select questions (MSQs) (select all that apply), full credit (4 points) was awarded only if all correct and no incorrect options were selected; no partial credit was given (totaling 40 points). This scoring system was designed to differentiate between levels of knowledge mastery, with a higher total score indicating a more comprehensive and applied understanding of PRO (15). The maximum score of 100 provides a clear, interpretable metric for knowledge assessment.

##### 2.6.1.2 Teaching Feedback Questionnaire

*Objective*: To qualitatively evaluate trainees’ subjective perceptions, attitudes, and satisfaction with the curriculum.

*Content and format*: The questionnaire contained items investigating interest in the course, satisfaction with the content, perceived helpfulness of PRO for medical learning, and attitudes toward the inclusion of PRO in medical intervention evaluations and education.

*Scoring system and basis*: Responses were collected on a 5-point Likert scale. The scoring criteria were explicitly defined as: 4 = “Strongly Agree,” 3 = “Somewhat Agree,” 2 = “Neither agree nor disagree,” 1 = “Disagree,” 0 = “Strongly Disagree” (14). The mean score for each item was calculated, with a higher score representing a more positive perception or stronger agreement. This method allows for the quantification of subjective feedback for comparative analysis.

#### 2.6.2 Administration of assessments

Both questionnaires were administered in a standardized, paper-based format. The pre-test was administered on the first day of the 2-month rotation, before any study-related activities began. The post-test knowledge assessment and the feedback questionnaire were administered on the final day of the same 2-month rotation, immediately after the completion of all rotational activities. The knowledge test and the feedback questionnaire were administered on-site immediately following the completion of the rotation. A dedicated staff member was present to distribute and collect the instruments promptly, ensuring a high response rate and protocol adherence. The feedback questionnaire was administered to both groups to maintain consistency, though we acknowledge potential interpretation bias for the control group, as discussed in the limitations.

### 2.7 Ethical consideration and remedial training for the control group

Upon completion of the post-intervention theoretical knowledge test, and prior to the administration of the Teaching Feedback Questionnaire, participants in the control group were provided with a remedial training session. This session was implemented as an ethical obligation to ensure that all trainees, regardless of their group assignment, had access to the core PRO educational content by the end of the study period.

The remedial training was a condensed, single session covering the essential concepts from the full PRO curriculum delivered to the experimental group, including an overview of PRO, PROMIS, OKS, and OHS. This session was deliberately designed as an informal curriculum—a one-time, didactic overview—to fulfill ethical obligations, thereby sharply contrasting with the multi-component, longitudinally delivered structured curriculum experienced by the experimental group. It is critical to note that this remedial training was conducted only after the control group had completed the final (post-test) knowledge assessment. This sequencing was strictly maintained to prevent contamination of the primary outcome data (the post-test knowledge scores used for between-group comparisons). The feedback questionnaire was then administered to both groups following this remedial session for the controls.

### 2.8 Statistical analysis

All statistical analyses were performed using SPSS software (Version 25.0, IBM Corp., Armonk, NY, United States). Continuous variables that were normally distributed are presented as mean ± standard deviation (SD). Categorical data are summarized as frequencies and percentages (*n*, %). The primary analyses included:

*Within-group comparisons*: Paired sample *t*-tests (two-tailed) were used to evaluate the improvement in PRO knowledge scores from pre-test to post-test within each group.
*Between-group comparisons*: Independent sample *t*-tests (two-tailed) were used to compare the post-test knowledge scores and the feedback questionnaire responses between the experimental and control groups. The threshold for statistical significance was set *a priori* at a *p*-value of less than 0.05 (*α* = 0.05).

## 3 Results

### 3.1 Participant characteristics

The study comprised two equal-sized groups (*n* = 25 each): the experimental group and the control group. Table 1 presents the detailed demographic and baseline characteristics of the participants. The groups were well-matched in terms of age, sex, training year distribution, and prior clinical experience in orthopedics (as measured by the number of completed orthopedic rotations prior to the study period). Crucially, there was no significant difference in the baseline PRO knowledge scores and the familiarity with PRO content between the groups, indicating comparable starting competency levels (Table 1).

### 3.2 Comparison of theoretical PRO knowledge test scores

All participants completed theoretical PRO knowledge test (Table 2). Initial testing revealed comparable baseline scores between the experimental group and the control group, with no statistically significant difference (*p* = 0.45). Following the standardized training, significant enhancements in PRO knowledge test scores were observed in both the experimental group (70.56 ± 5.89 vs. 40.96 ± 4.55, *p* < 0.001) and the control group (58.68 ± 4.72 vs. 42.04 ± 5.36, *p* < 0.001). Notably, the experimental group achieved significantly higher post-rotation scores compared to the control group (70.56 ± 5.89 vs. 58.68 ± 4.72, *p* < 0.001).

**Table 2:** Theoretical knowledge evaluation of resident trainees in the PRO teaching reform experimental group versus the traditional model teaching group.

Group	End-of-rotation examination (mean ± SD)	Pre-rotation examination (mean ± SD)	*t*	*p*
PRO teaching reform experimental group	70.56 ± 5.89	40.96 ± 4.55	18.94	<0.001^a^
Traditional model teaching group	58.68 ± 4.72	42.04 ± 5.36	18.80	<0.001^a^
*t*	7.869	−0.768		
*p*	<0.001^a^	0.446		

SD, standard deviation.

a Denotes a statistically significant difference (*p* < 0.05).

### 3.3 Application of patient PRO data in teaching

During the two-month rotation, trainees in the experimental group systematically collected pre-operative and one-month post-operative OKS or OHS data for approximately TKA and THA patients in clinical practice. These data were integrated into daily clinical teaching and case discussions, providing residents with rich, real-world examples to learn how to interpret PRO scores, track their changes over time, and understand their relationship with patients’ overall clinical status. As elaborated in the Discussion section, this process was a key component in achieving deeper knowledge and positive attitude shifts in the experimental group curriculum.

### 3.4 Results of the Teaching Feedback Questionnaire

**Figure 2:**
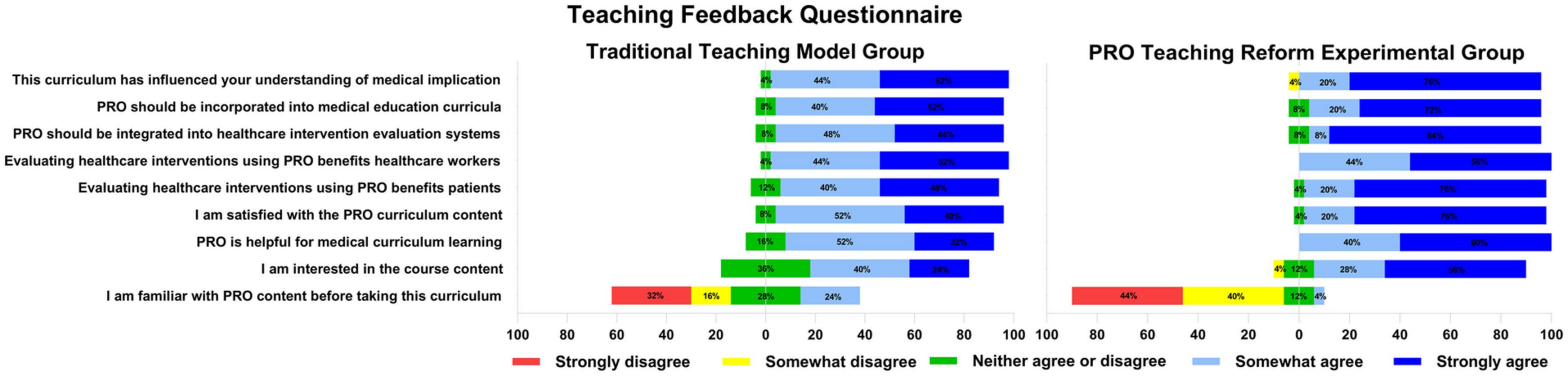
Comparison of Teaching Feedback Questionnaire responses between the PRO teaching reform experimental group and the traditional model teaching group. Back-to-back bar charts illustrate the percentage distribution of responses (from “Strongly disagree” to “Strongly agree”) for each survey item assessing trainees’ perceptions, attitudes, and satisfaction regarding the PRO curriculum.

According to the survey results, there were no significant differences between the two groups in the following aspects prior to the course: familiarity with PRO content; agreement on incorporating PRO into medical education curricula; recognition of the benefits of using PRO to evaluate medical interventions for healthcare professionals (Table 3). We reported the results of each item in Teaching Feedback Questionnaire in Figure 2. Compared with the control group, the experimental group showed significantly higher interest in the reformed curriculum (3.36 ± 0.86 vs. 2.88 ± 0.78, *p* = 0.04), higher satisfaction with the PRO course content (3.72 ± 0.54 vs. 3.32 ± 0.63, *p* = 0.02), higher agreement that PRO enhances medical education (3.60 ± 0.50 vs. 3.16 ± 0.69, *p* = 0.01). Furthermore, by integrating PRO theoretical knowledge into standardized orthopedic surgery training, the experimental group demonstrated stronger agreement on the benefits of PRO in evaluating medical interventions for patients (3.76 ± 0.44 vs. 3.44 ± 0.59, *p* = 0.03), and also showed stronger agreements on the importance of incorporating PRO into medical intervention assessment systems (3.76 ± 0.60 vs. 3.36 ± 0.64, *p* = 0.03). Given the integration of PRO theory into clinical practice in orthopedic surgery, the experimental group expressed significantly greater recognition (3.80 ± 0.41 vs. 3.48 ± 0.59, *p* = 0.03).

**Table 3:** The teaching feedback of resident trainees in the PRO teaching reform experimental group versus the traditional model teaching group.

Item	PRO teaching reform experimental group (mean ± SD)	Traditional model teaching group (mean ± SD)	*t*	*p*
I am familiar with PRO content before taking this curriculum	0.76 ± 0.83	1.00 ± 0.86	−1.00	0.322
I am interested in the course content	3.36 ± 0.86	2.88 ± 0.78	2.06	0.044^a^
PRO is helpful for medical curriculum learning	3.60 ± 0.50	3.16 ± 0.69	2.59	0.013^a^
I am satisfied with the PRO curriculum content	3.72 ± 0.54	3.32 ± 0.63	2.41	0.019^a^
Evaluating healthcare interventions using PRO benefits patients	3.76 ± 0.44	3.44 ± 0.59	2.20	0.033^a^
Evaluating healthcare interventions using PRO benefits healthcare workers	3.56 ± 0.51	3.52 ± 0.59	0.26	0.797
PRO should be integrated into healthcare intervention evaluation systems	3.76 ± 0.60	3.36 ± 0.64	2.29	0.026^a^
PRO should be incorporated into medical education curricula	3.64 ± 0.64	3.44 ± 0.65	1.10	0.277
This curriculum has influenced your understanding of medical implication	3.80 ± 0.41	3.48 ± 0.59	2.24	0.026^a^

SD, standard deviation, each item employs a 5-point Likert scale of 0 (very low) to 4 (very high).

a Denotes a statistically significant difference (*p* < 0.05).

## 4 Discussion

This pilot study found that integrating a structured PRO curriculum into orthopedic residency training significantly improved residents’ PRO knowledge and attitudes. While both groups gained knowledge, the experimental group showed greater improvement, highlighting the value of formal PRO education. At baseline, both groups performed poorly on the PRO knowledge assessment, indicating a general lack of prior exposure or instruction in this area. This systemic deficit can be directly traced to the current national training curriculum for orthopedic residency in China, which, upon review, does not include PRO education within its required competencies. Our findings, therefore, not only align with global reports but also highlight a curriculum-specific gap within this standardized training system (7). Our findings, consistent with Florentino et al. (7), confirm that this educational gap persists in the critical early stage of postgraduate surgical training. Although PRO is increasingly emphasized in clinical practice and health policy, it is not currently a requirement in the Liaison Committee on Medical Education (LCME) or Accreditation Council for Graduate Medical Education (ACGME) curriculum (16, 17).

Interestingly, the control group also showed substantial post-rotation improvement despite the absence of formal instruction. This suggests that clinical exposure in orthopedic surgery—where functional outcomes like pain reduction and range of motion are routinely tracked—can naturally promote familiarity with PRO-related concepts. However, the knowledge acquired through clinical experience alone appears unsystematic and less comprehensive, as evidenced by the significantly lower post-test scores of the control group compared to the intervention group (58.68 vs. 70.56, *p* < 0.001). This disparity underscores that a structured curriculum is essential for delivering a comprehensive and uniform understanding of PRO, covering both conceptual foundations and the application of specific instruments like PROMIS, OKS, and OHS. Residents in the experimental group participated in a full clinical cycle, from preoperative assessment using validated PRO scales to postoperative care and follow-up evaluation. This hands-on involvement not only reinforced theoretical knowledge but also enhanced their appreciation for patient-centered care. These trainees noted substantial variation in subjective patient experiences following similar surgical interventions, especially in terms of pain perception and functional recovery. Such observations stimulated critical thinking and encouraged the incorporation of both patient-reported and physician-reported outcomes in clinical decision-making (18, 19).

This shift in clinical perspective is significant. Furthermore, residents in the experimental group expressed a stronger belief in the value of PRO for patient evaluation (Table 3), suggesting that the training may foster an attitude more conducive to integrating subjective and objective data in clinical practice. The positive shift in attitudes among our trainees mirrors the outcomes observed by Florentino et al. (7), where a curriculum intervention led to increased student recognition of PRO’s importance. Our study extends this finding by demonstrating that even within a demanding surgical residency, a relatively brief intervention can effectively foster a more patient-centric mindset. These findings are consistent with international initiatives—such as a multicenter German cohort study—that emphasize involving medical students in real-world clinical research using PRO (20, 21). Orthopedic elective procedures primarily aim to improve quality of life (22). However, discrepancies often exist between physician expectations and patient experiences. Prior studies have noted that patients’ satisfaction and perceived benefit from surgery do not always align with clinical indicators (23, 24). Training residents to understand and value PRO may help bridge this gap, encouraging them to assess outcomes from the patients’ perspective and ultimately leading to more empathetic, evidence-informed care.

Feedback from both groups indicated broad recognition of PRO’s relevance in evaluating medical interventions. Even participants in the control group—who received no structured PRO instruction—supported the inclusion of PRO-related content in the curriculum, likely reflecting the implicit influence of clinical exposure during residency. These results are in line with previous studies suggesting that structured PRO training enhances learners’ clinical insight and supports broader implementation in medical education (7, 25).

First, the selection of OKS and OHS as focal PRO instruments was intentional, as they represent classic and commonly used tools in orthopedic arthroplasty. The primary aim was to equip residents with the fundamental principles and processes of PRO application through these exemplars. Consequently, the core outcomes assessed were the learners’ PRO knowledge level and attitudes, rather than a comprehensive evaluation of multiple PRO tools. Trainees in the experimental group engaged deeply with patient OKS/OHS data, which served as critical mediators for achieving educational objectives, such as understanding the variability of patient subjective experiences. The aggregate patient data, while central to the learning process, was not a pre-specified efficacy outcome measure for this educational study.

Second, the definition of PRO emphasizes the direct assessment of health status from the patient’s perspective. The educational innovation here was to operationalize this definition by having residents personally collect and interpret OKS/OHS—instruments designed to capture “patients’ subjective feelings/experiences.” The trainees’ reported observation of “substantial variation in subjective patient experiences” underscores how engagement with real patient-derived data, as opposed to theoretical or simulated cases, fostered a tangible understanding of PRO’s value. This experiential learning was fundamental in shifting attitudes toward patient-centered care. Future research should build on this foundation to examine downstream effects, such as the quality of PRO utilization in clinical practice and the long-term impact on patient care experiences following such educational interventions.

A pertinent consideration is the distinction between the structured curriculum received by the experimental group and the informal, remedial overview provided to the control group. Our pilot study was designed to evaluate the feasibility of integrating a structured PRO curriculum into the existing clinical training framework, which inherently coupled the curricular structure (modular, spaced learning) with clinical integration over a 2-month rotation. The control group’s exposure, in contrast, was a single, isolated session without integrated practice. A future experiment designed to strictly control for both total teaching time and clinical exposure duration—for instance, comparing a structured, integrated curriculum against a consolidated lecture of equal total length—would be invaluable to isolate the specific effect of the curricular structure itself. While our current design cannot definitively disentangle these factors, the significant gains observed in the experimental group strongly suggest that the structured, integrated approach is a feasible and potent strategy for PRO education. The positive outcomes likely stem from the synergistic effect of repeated theoretical exposure and immediate clinical application, a core component of our structured intervention.

While this study evaluated immediate educational outcomes, the integration of PRO into residency training may have important long-term implications for clinical practice. By establishing familiarity and positive attitudes early in training, such a curriculum could encourage the habitual use of PRO in future independent practice, contributing to more systematic outcome monitoring and patient-centered care models. Furthermore, the structured approach—combining concise didactics with supervised clinical application—demonstrates a scalable model that could be adapted to other surgical or procedural specialties where patient-reported experience and functional recovery are key outcomes, such as neurosurgery, plastic surgery, or interventional cardiology.

Also, future research should build upon these promising findings by employing longitudinal designs to determine whether improved knowledge and positive attitudes translate into sustained changes in clinical behavior, such as the routine integration of PRO into patient assessments in independent practice. Additionally, investigating the potential impact of such training on patient-clinician communication, shared decision-making, and long-term patient-reported outcome would further delineate the educational value chain.

### 4.1 Limitations

Several limitations should be acknowledged in this study. Firstly, given the lack of prior literature addressing this specific issue, the comparative analysis was unfeasible. The use of convenience sampling, along with the limited sample size and single-center design, may introduce bias into our results. Future research should expand the sample size, incorporate additional variables, and extend the study scope through a multi-center design. Second, we did not control for the potential acquisition of PRO knowledge from external sources during the study period, though the short duration of the intervention and the shared environment of both groups likely mitigated this risk. Third, the study instruments, though developed by expert consensus, were not psychometrically validated. The feedback questionnaire, in particular, utilized an agree-disagree response format (known to encourage acquiescence bias) and contained pre-course familiarity as well as the curriculum’s influence items that were susceptible to recall and interpretation bias, especially for the control group (26, 27). This limits the validity and generalizability of the feedback outcomes. Future studies should employ rigorously validated instruments to confirm our findings. Additionally, the Hawthorne effect could not be eliminated, as participants aware of the study’s educational focus might have exhibited performance bias. Future multicenter studies with longer observation periods, validated PRO metrics, and blinded outcome assessments are needed to confirm these preliminary results. Furthermore, as a preliminary feasibility study in education, we primarily focused on direct outcomes at the resident level. Future larger-scale or effectiveness studies could consider incorporating the quality and completeness of PRO data collected by residents in clinical practice, and even its potential impact on patient communication and shared decision-making into the evaluation framework, thereby more comprehensively delineating the educational value chain of such curricula.

## 5 Conclusion

This study demonstrates that integrating PRO into standardized orthopedic surgery residency training can significantly enhance resident engagement, deepen PRO-related knowledge, and foster the development of clinical reasoning skills. The curriculum not only encouraged the practical application of theoretical knowledge to clinical contexts but also improved residents’ understanding of patient-centered care and heightened their awareness of clinical outcomes beyond traditional metrics. By aligning with global trends in PRO implementation, this educational innovation provides a valuable framework for more comprehensive outcome evaluation and supports the continued reform and advancement of orthopedic education and curriculum development.

## Data Availability

The original contributions presented in the study are included in the article/Supplementary material, further inquiries can be directed to the corresponding authors.
