# Supplementary material for: Bridging the educational gap: a pilot study of integrating patient-reported outcome into orthopedic residency training
Source: Front Med (Lausanne). 2026 Jan 6;12:1635610. doi: 10.3389/fmed.2025.1635610 (PMC12815863; doi:10.3389/fmed.2025.1635610)
Supplement: Supplementary file 1 [file Data_Sheet_1.pdf]

Supplementary 1

Patient-Reported Outcome Theoretical Knowledge Evaluation

Ⅰ **Multiple Choice Questions（Choose the only correct answer to each question）**

1. The content of patient self-reported assessments includes

A: Diseases and symptoms

B: Physical examinations in medical records

C: Laboratory tests

D: Imaging tests

2. Which of the following is NOT a significance of integrating patient-reported outcomes (PROs) into clinical practice?

A: Improving patient satisfaction

B: Enhancing patients' quality of life

C: Facilitating doctor-patient communication

D: Increasing symptom detection and management

3. Compared to observer assessments, the advantages of patient self-reports do NOT include:

A: Detecting more overlooked symptoms

B: Focusing on patients' subjective experiences

C: Improving hospital economic benefits

D: Strengthening doctor-patient communication

4. Which scale/questionnaire is a disease-specific patient-reported outcome (PRO) assessment tool?

A: Pain Rating Questionnaire

B: Symptom Self-Assessment Scale

C: Anxiety Self-Assessment Scale

D: Knee Replacement Functional Pain Assessment Scale

5. The development and validation of the PROMIS tool do NOT include:

A: Framework and concept definition

B: Item pool formation and refinement

C: Clinical application

D: Large-scale sample testing

6. The conceptual framework of the U.S. PROMIS system does NOT include:

A: Physical health

B: Mental health

C: Social health

D: Patient satisfaction

7. Which of the following is NOT a disease-specific scale for knee joint evaluation?

A: Western Ontario and McMaster Universities Osteoarthritis Index (WOMAC)

B: Oxford Knee Score (OKS)

C: Knee Injury and Osteoarthritis Outcome Score (KOOS)

D: Visual Analogue Scale (VAS) for Pain

8. Which of the following is NOT a method for evaluating patient-reported outcomes (PROs)?

A: Patient self-administered questionnaires

B: Face-to-face qualitative interviews

C: Telephone interviews

D: Laboratory tests

9. The PROMIS Physical Function and Pain Interference scales include:

A: Computerized Adaptive Testing (CAT)

B: Disabilities of the Arm, Shoulder, and Hand (DASH)

C: Physical Function Assessment (PFA)

D: PROMIS Upper Extremity (PROMIS-UE)

10. Which of the following is NOT a traditional patient-reported outcome scale most commonly used in orthopedic surgery?

A: SF-36 Scale

B: Visual Analogue Scale (VAS)

C: PROMIS System

D: Short Form Musculoskeletal Function Assessment (s-MFA)

II **Multiple Answer Questions（Choose the correct answers to each question）**

11. Methods for collecting patient-reported outcomes (PROs) include:

A: Patient oral history

B: Questionnaire/Scale surveys

C: Patient interviews

D: Healthcare provider inquiries

12. Categories of patient-reported outcome (PRO) scales include:

A: Generic scales

B: Specialized scales

C: Disease-specific scales

D: Comprehensive scales

13. The content of patient-reported outcomes (PROs) includes:

A: Disease-related experiences

B: Symptom intensity and impact

C: Patient satisfaction and compliance

D: Patient reports on doctor-patient communication, clinical collaboration, and treatment/nursing care

14. The value of patient-reported outcomes (PROs) lies in:

A: Only the patient knows the true effect of treatment

B: Patient experiences reflect the comprehensive function and goals of treatment

C: Formal assessments are more reliable than informal interviews

D: Subjective patient experiences should align with objective test results

15. Advantages of patient-reported outcomes (PROs) over observer assessments include:

A: Comprehensiveness

B: Objectivity and authenticity

C: Reliability

D: Standardization

16. Trends in the use of patient-reported outcomes (PROs) in clinical practice:

A: Increasing focus on specific PRO evaluation targets

B: Growing adoption of digital PRO tools

C: Equal importance of PROs and objective test results

D: PROs becoming more patient-centered

17. Key features of the PROMIS system include:

A: Comparability

B: Scientific rigor

C: Flexibility

D: Inclusiveness

18. Patient-reported outcomes (PROs) refer to information entirely reported by patients about their:

A: Disease symptoms

B: Health status

C: Financial situation

D: Quality of life-related well-being

19. PRO content for knee replacement surgery patients includes:

A: Pain severity

B: Knee joint range of motion

C: Lower limb muscle strength and its impact on daily life

D: Satisfaction with surgical outcomes

20. Hip joint functional assessment scales include:

A: Oxford Hip Score (OHS)

B: Thompson Hip Score

C: Harris Hip Score

D: WOMAC Hip Function Scale

III **True/False Questions**

21. In clinical practice, health information reported by observers and patient-reported outcomes may yield conflicting results.

□ True □ False

22. Patient-reported outcomes (PROs), as a form of scale-based assessment, encompass a broader scope than health-related quality of life (HRQL). However, HRQL is an essential component of PROs and irreplaceable in assessing health status.

□ True □ False

23. Patient-reported outcomes must not be subject to interference by healthcare providers.

□ True □ False

24. PROMIS represents a novel paradigm for patient-reported measurement in orthopedics and still holds significant potential for application in domestic orthopedic practice.

□ True □ False

25. Disease-specific PRO assessment tools are more sensitive and accurate than generic PRO assessment tools.

□ True □ False

26. Manual scoring is the most recommended method for evaluating outcomes using the PROMIS tool.

☐ True ☐ False

27. When reviewing the background and rationale in clinical trial research protocols, it is critical to focus on the specific research questions related to PROs.

☐ True ☐ False

28. PROMIS can not only measure patient symptoms and function but also predict postoperative recovery capacity in orthopedic patients, helping surgeons set realistic expectations for surgical outcomes preoperatively.

☐ True ☐ False

29. The Minimal Clinically Important Difference (MCID) reliably reflects changes in treatment or care effectiveness. Recently, researchers have widely used MCID to analyze and objectively interpret post-treatment PRO changes, providing critical evidence for clinical interventions or treatment selection.

☐ True ☐ False

30. Every domain of PROMIS employs dynamic Computerized Adaptive Testing (CAT) to reduce patient burden. Integrating PROMIS CAT into hospital electronic health record (EHR) systems enables real-time monitoring of patient symptoms and functional status.

☐ True ☐ False
