# Supplementary material for: Bridging the educational gap: a pilot study of integrating patient-reported outcome into orthopedic residency training
Source: Front Med (Lausanne). 2026 Jan 6;12:1635610. doi: 10.3389/fmed.2025.1635610 (PMC12815863; doi:10.3389/fmed.2025.1635610)
Supplement: Supplementary file 2 [file Data_Sheet_2.pdf]

Supplementary 2

Teaching Feedback Questionnaire on Patient-Reported Outcome (PRO) Curriculum

1. I am familiar with PRO content before taking this curriculum	**Strongly Disagree**	0	1	2	3	4	**Strongly Agree**
2. I am interested in the course content	**Strongly Disagree**	0	1	2	3	4	**Strongly Agree**
3. PRO is helpful for medical curriculum learning	**Strongly Disagree**	0	1	2	3	4	**Strongly Agree**
4. I am satisfied with the PRO curriculum content	**Strongly Disagree**	0	1	2	3	4	**Strongly Agree**
5. Evaluating healthcare interventions using PRO benefits patients	**Strongly Disagree**	0	1	2	3	4	**Strongly Agree**
6. Evaluating healthcare interventions using PRO benefits healthcare workers	**Strongly Disagree**	0	1	2	3	4	**Strongly Agree**
7. PRO should be integrated into healthcare intervention evaluation systems	**Strongly Disagree**	0	1	2	3	4	**Strongly Agree**
8. PRO should be incorporated into medical education curricula	**Strongly Disagree**	0	1	2	3	4	**Strongly Agree**
9. This curriculum has influenced your understanding of medical implication	**Strongly Disagree**	0	1	2	3	4	**Strongly Agree**

Additional suggestions
